# Developing a societal impact evaluation framework for sustainable European University Alliances

**DOI:** 10.1038/s41598-024-63933-9

**Published:** 2024-06-06

**Authors:** Laura Corazza, Francesco Marengo, Daniel Torchia, Massimo Sargiacomo

**Affiliations:** 1 Department of Management, University of Turin, Turin, Italy; 2 Department of Business and Law, University of Milano-Bicocca, Milan, Italy; 3 Department of Management and Business Administration, University ‘G. d’Annunzio’, Chieti-Pescara, Italy

**Keywords:** Socioeconomic scenarios, Sustainability

## Abstract

European University alliances, formally introduced in 2019, are rapidly expanding, as more than 400 million euros have been dedicated in 2023 by the European Commission to foster international collaborations to promote new forms of development within and beyond university communities. By undertaking interventionist research on UNITA – *Universitas Montium*, one of the largest European alliances, representing 160.000 students, this paper aims to illustrate how a university alliance is tasked with developing an internal assessment methodology to account for the societal benefits created by the project for the academic and civil communities. The elaboration of the assessment tool to assess the contribution to higher education and societal sustainable communities has brought researchers to discover etic and emic implications, revealing the existence of an accountability layer in which the international alliance directly engages with rural and mountain communities in marginalized areas. This research marks a significant advancement in the field of higher education sustainability, providing both a novel analytical perspective on the benefits of university alliances for the development of local sustainable communities and a methodological tool for their assessment.

## Introduction

Universities in Europe are gaining traction as new players capable of steering the development of new policies in the research and educational area and contributing to the creation of sustainable communities^1,2^. It is therefore unsurprising that the European Union has placed a lot of emphasis on higher education and research as drivers for sustainability development. On top of this, to get closer to achieving the persistent idea of creating European universities^3^, the establishment of European University Alliances (EUAs) plays an important role. The European Commission (EC) describes EUAs as ‘transnational alliances that will become the universities of the future, promoting European values and identity, and revolutionizing the quality and competitiveness of European higher education’^4^. Looking at what has been achieved so far, the Policy Department for Structural and Cohesion Policies of the European Parliament^4^ lists increased European cohesion, higher social engagement, and more readiness to market needs as benefits of EUAs.

The European Universities Initiative (EUI), promoted by the EC and formally introduced in 2019, aims at enhancing cross-border collaboration among higher education institutions through formal alliances, benefitting from long-term integration of activities, or even institutions^5^. Following two calls, by 2020, 41 alliances were formed, involving 279 universities in 32 European countries, with assigned budgets, clear goals, and expected deliverables. As we progress toward the concept of the European university, and considering the attention that the EC is paying to this, the number of alliances is expected to further grow and to realize impacts that go beyond higher education^4^. Nonetheless, the concept of a European university could imply a variety and diversity of options in the governance mechanisms and in the development of institutional strategies^6^, and perhaps surprisingly, to date much remains to be discovered on how universities influence policymaking at the European level, and on how their impact can be properly assessed and evaluated. The limited scholarly engagement on these matters goes against the growing number of alliances established in Europe^7^. In line with this rising trend, there is no more time to waste on the development of proper social assessment tools for EUAs, beyond the focus on financial and economic performance indicators^8,9^.

Despite the increasing interest in promoting principles of university social responsibility^10^ and universities’ accountability^11^, the concept of social impact in universities remains not easy to address. Social impacts are intended as any consequence of a project or intervention directly or indirectly affecting people, whose measurement and assessment is part of a process of impact management^12^. When it comes to the social impacts generated by universities, they go beyond the missions of teaching, research, and societal outreach, to include impacts generated on the economic, entrepreneurial, institutional, political systems, also privileging the impacts on the production and transferal of knowledge. Given the several different types of activities conducted by universities, many studies focus on the social impacts generated by the main missions of teaching and researching^13^, and residually on those third mission activities of societal outreach^14^. While the impacts of increasing access towards global literacy, and especially access to higher education teaching activities, have been analyzed in various studies since the second half of the twentieth century^15^, the social impacts of university research have been subject to increasing academic attention in the last couple of decades^16^.

The study of the impact generated by universities is a highly cross-cutting issue that concerns both the functioning of university institutions and the generating force of local development that they can bring^17^. For this reason, universities are seen through their role as anchor institutions, where alongside economic and societal development there is a solid institutional presence of universities as an inseparable part of the local system^14^. Therefore, addressing the question of the impacts generated by a university is a complex issue, involving factors such as the size and location of the university campuses^18^, the link between the university and the territory^19^, how it pursues its missions and vocation, how it interprets its role for sustainable development^20^ as well as the mechanisms of communication and accountability^21,22^ existing between the university and the territory^10,23,24^.

In some studies, addressing the theme of societal benefits generated by universities^25,26^, and specifically in Europe^27,28^, those activities beyond teaching and research in which universities engage directly with society for the public interest fall under the category of third mission^29^. However, even though the third mission activities include the promotion of sustainable development among the local communities^30^, the term third mission still lacks a clear definition. Both direct university engagement in social and cultural university life, as well as research and teaching-related activities such as technology transfer, innovation, and lifelong learning fall under the categorization of the third mission^29^. Therefore, it becomes rather challenging to determine whether the societal effects arise specifically from third-mission activities, or in conjunction with teaching and research projects^31^.

Considering the vastity of universities’ activities and their respective different areas of impact, it is challenging to comprehensively analyze all their impacts on society. An attempt in this direction can be drawn from universities’ sustainability accounting practices, which contribute to proving the impacts and contribution to sustainability by higher education institutions through the disclosure of information regarding social, environmental, and economic performances^32^. Despite its usefulness in providing information on sustainability-related issues^33–35^, universities are not as thorough in reporting the outcome of their initiatives as for-profit organizations, as they report quite limited information, mainly concerning the environmental and economic dimensions, over the social aspects^32^. This leaves considerable room for research into how to properly identify and assess social impacts of higher education institutions^29^.

This study adopts an interventionist approach^36–41^ to develop a framework for assessing the societal impacts of the UNITA *Universitas Montium* (hereinafter UNITA) alliance, set up in 2020 as part of the European University Call, to create (and strengthen) international sustainable communities in marginalized rural and mountain areas. The alliance started with six member universities, and it expanded in 2023 to twelve.

The intervention lasted for a year and entailed deep engagement with various stakeholders of the UNITA alliance, leading to the development and first testing of a series of socio-economic indicators, directly linked to 8 work packages of the alliance. As per the tradition of interventionist research, the study contributes to both theory and practice, acting on both the *etic* and *emic* domains. First, it contributes to the literature in social and environmental accounting and university, which so far has only partially explored the societal impacts of university, and from a limited perspective (e.g. by looking at one of their missions, rather than in an ecosystemic way). Second, it provides a fresh look at university alliances beyond the organizational point of view, to include accountability and societal impact assessment measures to evaluate their contribution to promoting sustainable communities. Third, the study contributes to practice, by offering a method to identify and assess such impacts that can be adapted to other transversal university projects, especially those involving collaborations, like formal and informal alliances.

## Materials and methods

### Study area: UNITA Universitas Montium

UNITA *Universitas Montium* is a coalition of universities located in different countries, which when this study was conducted, accounted for 6 universities in 5 different countries (namely Portugal, Spain, France, Italy, and Romania) for a total of 160,000 students and 13,000 staff members. Its name, UNITA, recalls the strong connections and sense of belonging of member universities. With the addition of 6 new partners at the end of 2023, the students involved have grown to 248,000 and the staff members to 20,000. The alliance also has 35 associated partners, including national and international organizations, local authorities, other universities, and representatives of the socio-economic sphere.

UNITA was established in 2020 after being allocated funding from the European University Call under the program Erasmus + *Key Action 2: Cooperation for innovation and exchange of good practices* in European Universities. UNITA was one of the 24 total EUAs selected to share the total budget of €120 million. UNITA’s members have a shared commitment to deliver targeted education interventions and research results, which are aimed at driving community and socio-economic development of the territories they are in, but within a European dimension^42^. Furthermore, the six founding members are also drawn together by three fundamental characteristics. First, they are situated in rural mountain regions: Serra da Estrela, Pyrenees, Alps, and Banat (Timisoara). Second, they are positioned in cross-border areas of Southern, Central, and Eastern Europe, ecosystems that face related challenges. Third, they are in neo-Latin speaking countries, intending to strengthen linguistic diversity and mutual understanding, and thus promoting the use of languages besides English. Cultural commonalities and a strong shared vision not only enhance the alliance’s identity but are also key to leading the activities towards common goals, which might contribute to the long-term success and stability of the alliance^43^. The operations of the alliance were led by 8 Work Packages (WPs) task forces, each in charge of achieving a specific objective within the alliance (see “Supplementary Information” for insights on UNITA’s member universities, institutional structure, governance processes, mission, objectives, and values).

In July 2023, the UNITA University Alliance was awarded a second round of funding equal to 14 million euros to further expand its activities and strengthen the alliance. With this in mind, the next section is dedicated to Interventionist Research as the main method used in this study.

### Interventionist research

Interventionist research (IVR) is a ‘research methodology based on case study research, whereby researchers involve themselves in working directly with managers in organizations to solve real-world problems by deploying theory for designing and implementing solutions through interventions and analyzing the results from both a theoretical and practice perspective”^44^.

Finding its roots in the work of social psychologist Lewin^44–46^, IVR is a rather popular method in accounting and managerial studies and should therefore not be mistaken with methodologies used in other fields of studies^46^, such as *clinical research*, *design science*, or even closer ones like *action research*, *action science,* and *constructive research*^44^. Therefore, prominent researchers^44,46,47^ posit that IVR should be considered a stand-alone methodology, whose key characteristic is the combination of applications and contributions to theory (within the *etic* domain) with a strong intervention to the object of study, which should lead to a contribution to practice (within the *emic* domain). To achieve this, researchers are actively seeking to engage with (and solve) real-world problems, rather than simply studying how such issues are dealt with by managers^39^.

Consequently, and somehow similarly to action research, in IVR there is a high level of interaction between the researcher and the research object, while striving to maintain objectivity and neutrality as much as possible. The closeness to the organization that IVR researchers acquire, allows them to study the matters at play from within, giving strength to the intervention^48^. Empirical IVR research is conducted in vivo rather than *ex-post*, so that the analysis follows the unfolding of the events, stressing how and why events occur^46^. This further differentiates IVR from other types of case study research, with the latter more commonly applying an *ex-post*, or even longitudinal approach to analysis.

The team of researchers was structured in a way to have both a representation directly involved in the intervention and an external component able to supervise the study without organizational bias. It should be noted that the dialogic component of IVR between researchers and practitioners allows for the co-construction of meaning and the metacognitive attribution of meaning to be attributed to what is called an accounting device^49^. It turns out to be crucial, then, that the meaning-making process is built *in itinere*, through constant collaborative discussion and dialogue in key situations apt to undertake a logical exploration of the adoption of a new accounting tool, rather than being a mechanistic process^41^.

**Figure 1:**
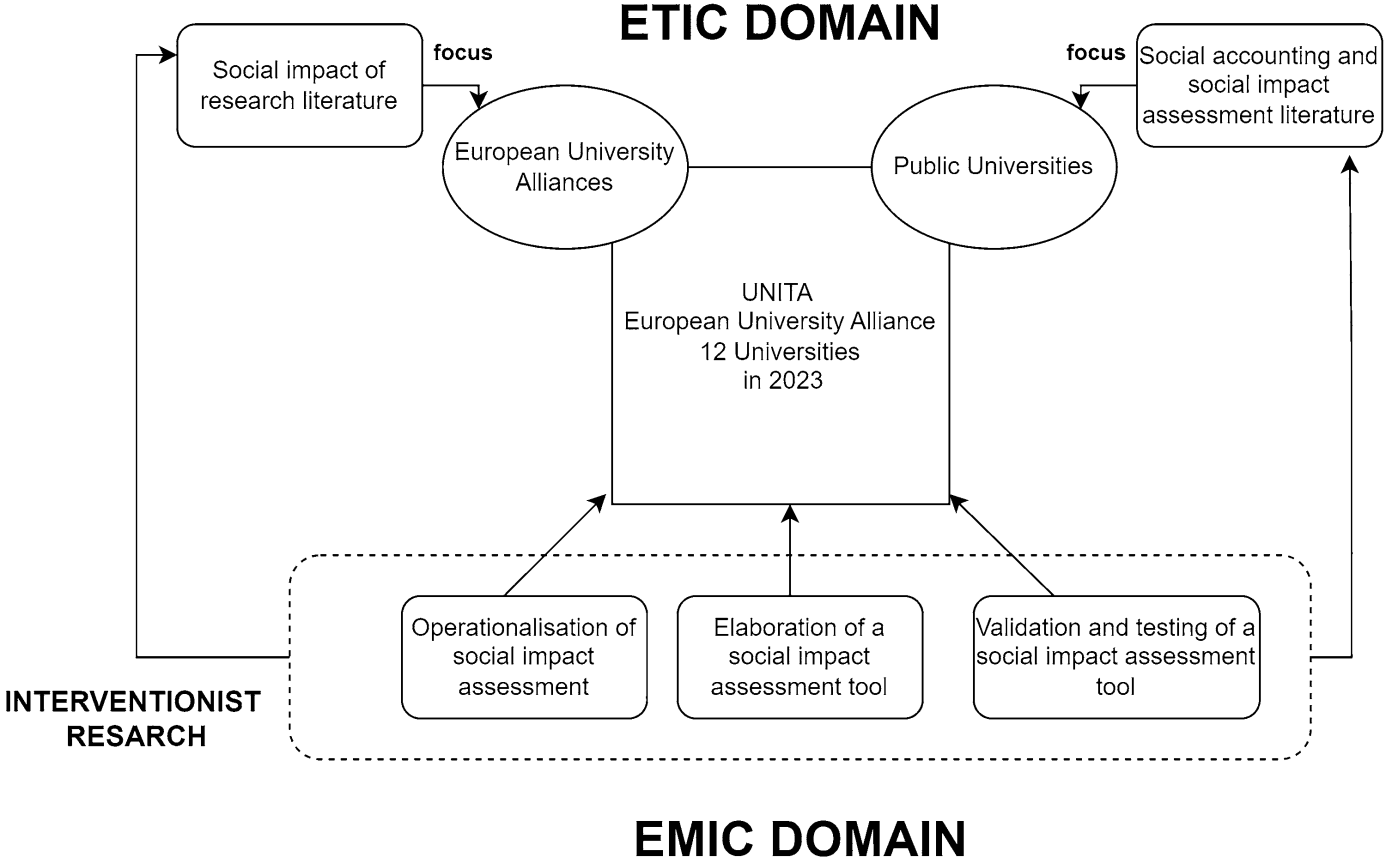
European citizenship, avoiding marginalization.

With this intent, this study also presents the creative application of IVR, as this study could be helpful not only in measuring and assessing the societal impacts of a university alliance but also in mobilizing the potentialities of accounting instruments to create sustainable communities by enhancing sub-politics for higher level values^50^, such as European citizenship, avoiding marginalization, and fostering social inclusion (Fig. 1).

### Research design

The interventionist approach applied to this research adopts the three-stage model presented by Jansen^51^, with stage 1 being the intervention design, stage 2 its implementation and stage 3 its evaluation. The intervention took place from May 2022 to May 2023.

Stage 1 started with preparatory meetings with the University of Turin’s (henceforth UNITO) Rector UNITA’s governance, giving the green light to the project. With UNITA entering its third year of operations and the final year of the initial funding granted, the organization was working on the upcoming call. In light of this, UNITA needed to show positive societal impacts, in order to obtain new European funding and give a long-term perspective to the alliance. Then, this stage split into the e*mic* and e*tic* domains: the former consisting of the analysis of UNITA’s internal documents (e.g. Project Proposal, Mid-Term Progress Report, WPs and GANTT charts), structure and operations (the activities related to the 8 WPs), and the latter concerning the analysis of academic literature on universities’ main missions (teaching, research and third mission) and their societal impacts.

Stage 2 includes the application and presentation of the method, following a process-based approach, revolving around pathways to impacts. First, the identification of the impacts follows a pathway structure that takes into account the early effects of UNITA’s tasks, ending with longer-term impacts in a causal logic. Second, through the identification and involvement of stakeholders in the creation and selection of societal impact indicators for measuring such impacts.

Stage 3, the final one, concerns the evaluation of the intervention, and it follows an outcome-based approach. This stage entails the evaluation, validation, and eventually the re-elaboration of the indicators, which are checked and revised if needed. This step, again validated by different categories of stakeholders (for instance, the WP task force and other stakeholders involved in the activities) also entails identifying new indicators and/or eliminating those that proved to be irrelevant.

**Figure 2:**
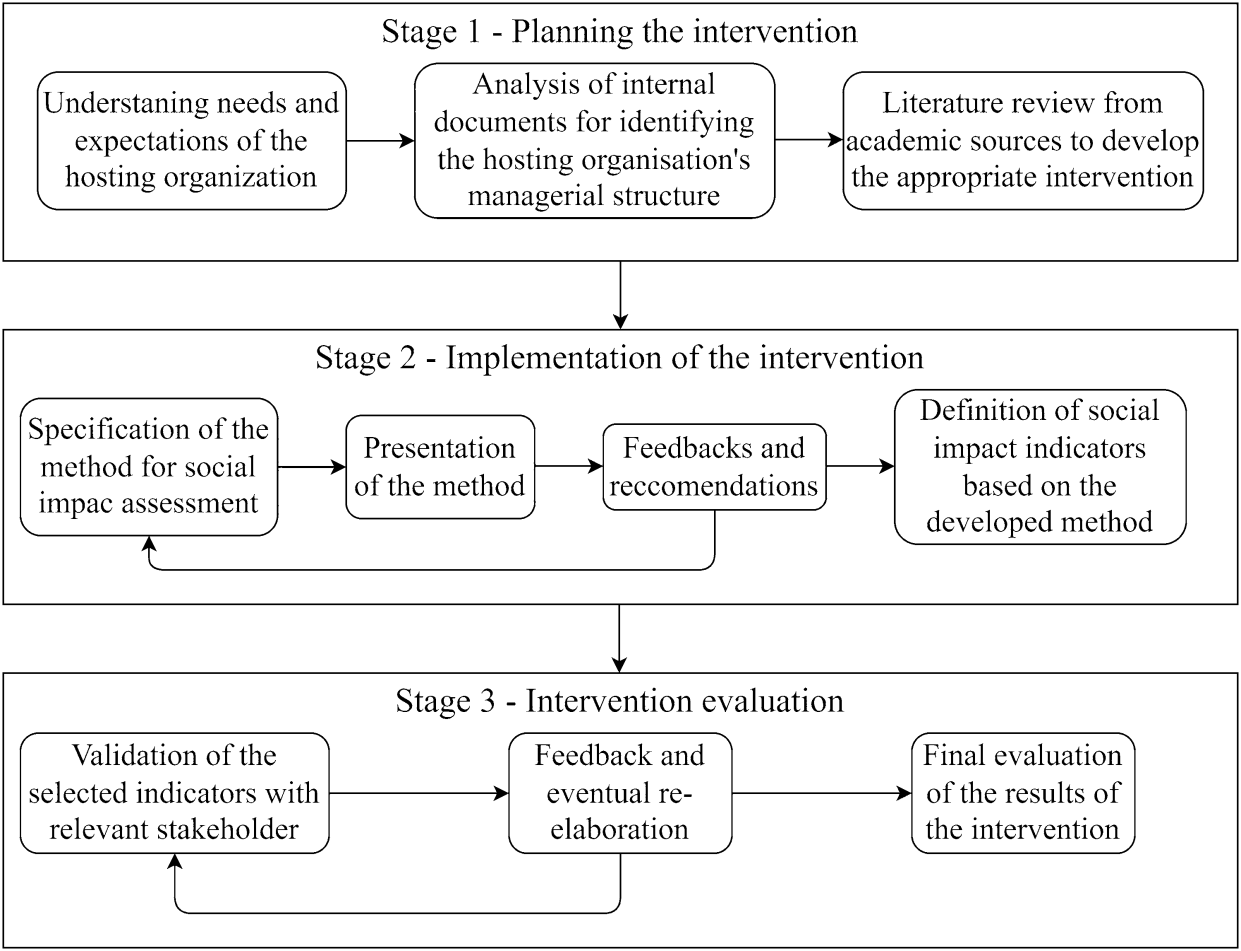
Methodological steps of the intervention.

The three stages and the respective methodological steps of the intervention are visually represented in Fig. 2. Aside from the analysis of the organizational internal documents and the literature review of academic sources to inform the development of the intervention, all the methodological steps have been informed by a total of 23 interviews and focus meetings with academics, executive managers, team leaders, and administrative staff working for the hosting organization (UNITA). Meeting minutes, recordings, email exchanges, internal reporting, project progress reports, and websites were used as incidental but essential documentary sources in defining appropriate contextualization. Globally, this study has led to interactions with more than 80 participants, whose involvement in the project has been longitudinal^52^. To ensure that the data collected is significant for developing a method to assess the societal impacts of the alliance, participants in the study were selected to represent all Work Packages responsible for implementing UNITA’s project activities, all governance bodies, and all administrative units of the alliance. This comprehensive approach ensures that all the alliance’s activities are addressed and considered in the development of a thorough impact assessment process. Specific information on the qualitative data collected during the interviews and focus groups with study participants is detailed in the data collection section of the “Supplementary Information” annex.

## Results

### Interventionist case study

As sometimes happens in IVR, the collaboration with the hosting organization for the development of the intervention was directly sought after by the governance members of the organization themselves^39^. One of the authors, as well as being a direct participant in the intervention, has been in charge for the previous 8 years of elaborating the sustainability report of the University of Turin (in brief UNITO), which is the leading member of UNITA (the Alliance). Acknowledging the work done on the sustainability report, and particularly regarding the section on social sustainability, the rector of UNITO directly contacted one of the authors to develop a method to identify, measure and evaluate the societal impacts of the activities conducted by the alliance. The IVR process started with this first meeting with the rector. The intervention consisted of the development and application of a method for the identification and measurement of UNITA’s societal impacts.

### Intervention design

The first phase of the intervention, which culminated in the design of the intervention, took place from May to mid-November 2022. During this time, the work conducted and the meetings with the members of the hosting organization were focused on gathering information for the development and design of the intervention. This process focused on three main aspects:

- Understanding the needs, expectations, and problematic aspects of the intervention for the hosting organization.
- Analyzing the organizational structure and nature of the activities and operations of UNITA.
- Drawing theoretical insights and available methodologies from the academic literature for the assessment and measurement of societal impacts in the context of higher education institutions.

As said above, at the beginning of this IVR project, UNITA was entering its third year of operations, with the initial funding set to expire by the end of that year. UNITA members were already working on formulating a proposal for the upcoming second round of the 'European University' call. Consequently, it was crucial to devise concrete ways of showcasing its societal contributions during its initial years, especially in those rural and marginalized communities that are at the core of the alliance’s vision, to secure new grants and ensure the sustained existence and progress of the alliance. In particular, the needs and expectations were addressed during the first meetings, including assessing the long-lasting effects of the alliance on member universities and the influence on societies and communities at different levels, from rural to supranational. Therefore, through the intervention, it must be developed a societal impact tool capable of answering those questions, and to demonstrate the stable long-term transformative impacts of the activities conducted by the alliance as a form of dialogic accountability^53^.

Internal documents and archives, as well as a focus meeting with UNITA’s executive coordinator, have been the main analytical tools to grasp the organizational structure of the alliance, and the nature of its activities and operations. Specifically, operations were overseen by eight WPs mirroring the alliance's eight primary objectives, and by tasks, developed with the intent of matching one or more of the general objectives and mission of the alliance. Finally, the third part of the design phase focused on the analysis of the literature, looking for elements (or a framework) for the development of a method to assess and analyze the societal impacts of UNITA. Finding a way to rationally individuate and measure the impacts of such a wide range of activities has appeared to be a challenge for various alliances (not only for UNITA). Impact Pathways^54^ and Theory of Change (ToC)^55^ have been chosen, as they are applicable to different contexts and can be linked with the UNITA’s organizational structure of the activities.

**Figure 3:**
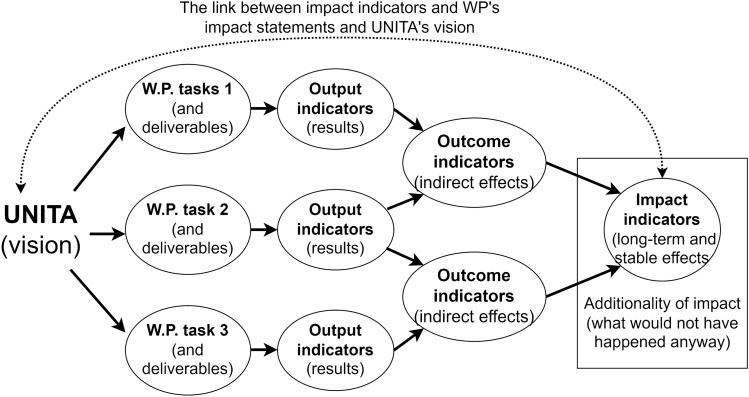
Participants of internships organized in rural area.

The resulting method, which works in accordance with ToC and impact pathways principles, is based on the selection of indicators for output, outcome, and impact based on the effects generated by the completion of the tasks of each WP, enabling the identification of impact pathways and providing a means to measure and assess them. Specifically, output indicators measure the direct effects (short-term) produced by an activity, such as the number of participants if the task consisted of organizing a workshop. Then, outcome indicators measure the indirect mid-term effect generated by an activity such as, for instance, the gain in social capital and connections of workshop participants. Impact indicators measure indirect, long-term transformative impacts generated by the activity(es), like higher employability experienced by participants of internships organized in rural areas. This process is explained in Fig. 3.

Finally, two more crucial criteria to identify impact indicators are followed: ensuring that the impact statements are aligned with UNITA’s vision, and that the impacts are directly attributable to the activity of UNITA. With the aim of capturing the heterogeneous nature of societal impact, the method does not pose limitations on the types of selectable indicators, which can therefore be qualitative, quantitative, or mixed.

### Implementation of the intervention: application and presentation of the method

In November and December 2022, the application of the method (for the identification of the indicators) and presentation took place in an iterative process. During the meetings, the team presented the method and provided updates on the selected indicators, gathering feedback and advice, functional to prepare for the following meeting. This phase helped the team in getting new information on how to apply the method within UNITA, and to present the preliminary results on the main areas of impact of the alliance. It also became apparent that it was not necessary to develop an output, outcome, and impact indicator for each task, because not all of them necessarily lead to long-term impacts, especially considering that some tasks are conducted progressively, so that they jointly contribute to reaching a common objective over time.

Along with the possibility that multiple tasks lead to only one impact indicator, it has also been found that some impacts (and respective indicators) may not be ascribable to any particular task (or group of tasks), which can happen when they result from activities that were not initially intended to take place, or when the impact can be considered the result of a WP as a whole. For this reason, general (namely not task or group of task-specific) indicators have also been included, paying particular attention to developing outcome and impact indicators to maintain the logical coherence of the progressive steps toward impact, with reference to each activity conducted. Finally, it was recommended to make sure that different indicators should not, even partly, measure the same impact. This recommendation is of particular importance, considering that some tasks contribute to the same goal.

The second type of findings is related to four main areas of societal impact that have arisen from the development of the indicators and by engaging with stakeholders at all phases of the intervention: university organizational impacts; new forms of learning and mobility opportunities impacting the quality of universities programs; impacts on the civil society and communities at multiple geographical scales; and economic and financial impacts.

### Intervention evaluation: validation and feedback on the selected indicators

This final phase of the intervention took place from January to May 2023, and offered two main contributions, the first being related to preliminary insights on the societal impact performance of UNITA and the second by further unfolding the peculiarities of UNITA’s impact. The main actors could work on a shared Excel spreadsheet, where per each WP a series of inputs, activities, outputs, outcomes, and impacts, have been listed and associated to the core areas of impact of the alliance. During that activity, the researchers engaged the WP leaders or any other representative in a continuous discussion regarding the sense, and the attribution of sense to the KPIs that researchers have previously drafted. Comments and notes have been collected by researchers in order to provide feedback to the participants.

Following a period of debate and discussion, the researchers have identified two specific contributions. The first, emerging from the direct application of the method, and concerning UNITA’s evaluation, is that different lines of activities may differ in terms of the impact produced, with some underperforming and others exceeding the expectations, producing positive results that were not even initially planned for. The second is instead related to the individualizations of UNITA’s specific impacts. While the areas of impact individuated in the previous phase, in line with the findings of previous studies^4^, the European University Initiative objectives, and the feedback of relevant actors engaged in this intervention are generally common to all EUAs, there are specific ways in which UNITA has contributed to those areas of impact, especially regarding impacts on civil society. In fact, thanks to the activation of innovative educational offers such as Rural Erasmus internships and public engagement activities in rural areas, the UNITA alliance has been able to enrich rural communities by creating a direct link between the international and local levels.

These specific ways by which UNITA has generated positive impacts are shown in Table 1, which shows the case-specific impacts related to the general ones and connected with the respective WPs that have contributed to their achievement.

**Table 1:** Identification of the impact areas specific to the UNITA Alliance.

Area of impact	Case-specific operations
University organizational structures	UNITA has been able to legally constitute itself in a legal form recognized by European law. UNITA members have also acted as pioneers in institutionalizing cooperation between different university alliances
Learning and mobility opportunities	UNITA has developed an innovative form of mobility: Rural mobility, allowing students to experience traineeship mobility in rural settings abroad. Moreover, the alliance, through inter-comprehension, has incentivized new forms of inclusive student-centered and digital learning pedagogies by training the teachers of member universities
Civil society	UNITA, through the implementation of entrepreneurship and empowerment programs with local organizations, has promoted the development of rural and mountain areas
Economic and Financial	UNITA has been able to activate and finance spin-off projects such as RE-UNITA (focused on research), INNOUNITA (focused on innovation and entrepreneurship) and GEMINAE (focused on establishing institutional collaborations with universities situated in Romance-speaking countries in other continents)

From the analysis of the case-specific impacts, it emerges how impacts of university alliances are tailored to the peculiarities of its member universities, which are in this case all situated in Romance language-speaking countries and rural or mountain cross-border areas. These common characteristics lead to unique impacts on society at different geographical levels, from the impact on the development of local marginalized communities to contributions at the international level on shaping the European higher education landscape. As such, the activity of societal impact assessment could not be fully standardized, but a process of co-creation and co-developing of KPIs is essential in grasping the relative knowledge of the impacts envisioned and those achieved.

Especially during the third phase, it has become very clear that acquiring an impact assessment mindset calls for a metacognitive habit of pushing boundaries and multi-stakeholder thinking that initially was not familiar to many participants. The idea that accounting for impacts goes beyond the legal boundary of the university has been seen as a problem that the university alliance has contributed to solving, as a protected environment in which such a kind of experimental accounting could be tested, (see Table 2).

**Table 2:** Extract of the societal impact assessment framework.

WP4 objectives	Tasks	Deliverables	Outputs	Outcomes	Impacts
Focusing research on the territories	Identifying actors in the three main areas	A cartography of research in the three thematic areas	Cartography of research: n. of researchers and research projects in the three thematic areas	N. of researchers and stakeholders integrated in the R&I Thematic Hubs:	
	Designing models for UNITA R&I Hubs	An operational model for the R&I Hubs (including Action Groups)	Description of the operational model of the R&I Hubs	N. of R&I projects born from the hubs	N. of patents born from the R&I Hubs
	Implementing UNITA R&I Hubs connecting all stakeholders	A strategic five-year R&I plan in the three thematic areas	N. of accesses to the cartography	Description of activities led by the research hubs	N. of stable formal agreements, eventual startups and/or spinoffs (not intended in the initial call) with external organizations in the three thematic areas
Connecting research and learning	Introducing bachelors’ students to research through micro-credentials, when possible, with short stays in the rural and mountains territories	Micro-credentials in the three thematic areas for bachelor students	N. of micro-credentials issued in the three thematic areas		Total n. of graduated students who obtained micro-credential in the three thematic areas or who participated in summer/winter schools, showing the (expected) increase over time
	Fostering research-based master education through summer / winter schools	Student reports on summer / winter schools for master students	Results of the surveys on the winter/summer schools	N. of students who applied to the winter/summer schools divided into their respective field of study	
	Enhancing links among the UNITA PhD programs	Report on personalized doctoral itineraries in the three thematic areas	N. of co-toutelle activated	N. of visits (transfers) of the UNITA PhD students	
Energizing the territories	Transferring research results for life-long learners	Micro-credentials in the three thematic areas	N. of micro-credentials issued	Results of surveys on the life-long learning courses on personal enrichment	N. of interns who started working for the hosting organization after the internship
	Connecting with stakeholders	Database of R&I internship offers	N. of the internship offers collected in the database	N. of projects led by external organizations asking UNITA (or its members) to become secondary partner in the areas of CE, CH, RE	Total n. of issued micro-credentials inserted in the cv/LinkedIn
	Enhancing entrepreneurship and employment	Catalogue of joint research projects in the three thematic areas for the development of rural and mountain territories	N. of downloads/accesses to the catalogue of projects	N. of local actors engaged in R&I hubs activities	
Additional indicators not traceable to a specific task			Matching events: n. of people participating in the matching events	Total n. of carried out UNITA traineeship	

As a result of the engagement process, several other impacts have emerged, such as the importance of the localization of the impacts and the impacts generated by activities of capacity building between the alliance partners. Localizing impacts helps acknowledging that a university alliance pushes the boundaries of the paradigm of the silos/closed university in places where the university is usually not present, such as in rural and internal mountain areas, even with short summer schools or workshops. Understanding that “place” could be important to emphasize impacts and help promoting sustainable development in marginalized communities, has been a radical shift brought by the alliance, which has required breaking traditional habits of closeness. This has been also evident in the capacity building and service innovations that the alliance has generated internally to UNITO and, by derivation, to all the partners involved. For instance, the adoption of a new ontology of processes and methods to account for participants coming from other countries has required the complete re-design of the status quo (in other words, before the introduction of the accountability tool), while nudging the university citizenry in increasing the number of services that are shared among the partners in the alliances (as such, identifying new roles and responsibilities among the different university departments). Finally, the geography of impacts has directly contributed to the development of the Europeanist sentiment through the abatement of intercultural stigma by developing intercomprehension labs.

## Discussion and conclusion

The first aim of this paper has been to investigate the societal beneficial impacts generated by university alliances and devise a means to assess and evaluate them. The purpose of universities has dramatically changed alongside the evolution of the external environment, especially after the COVID-19 pandemic^56^, and it is becoming even more complex. Consequently, what is expected from higher education institutions should be progressively redesigned to match these new needs^23^. Without questioning the role of universities as anchor place-based institutions^17^, it should be noted that the need for new services, innovations in teaching and learning, and new challenges brought by digitalization, require answers that universities must provide, which should also be innovative, inclusive, and sustainable.

The case of EUAs is becoming an important phenomenon, and studying the impacts of such university alliances brings new challenges in the research field of the societal impact of university research, both in terms of measurement and accountability duties. UNITA, a double-funded research alliance has been used as a focal case, as it will implement a process of accounting for societal impact, for the creation of long-term sustainable communities. This research contributes to the ever-evolving field of interventionist research in social and environmental accounting. This field is conquering novel attention in accounting studies (but not exclusively) in the last few years, not only for its ability to bring fresh insights in terms of operationalization of accounting in real settings^39,44^, but also for its contribution to the acceptance of this research method with a critical intent^40,52,57^. As such, the case of the UNITA alliance could be seen as material and experimental, as in this paper several interplays between the fields of *etic* and *emic* have been presented.

Within the *etic* domain, this paper contributes to the field of social impact assessment of university research. Drawing back to the literature on accountability^11,58^ and social impact assessment, recent discussions highlights the inadequacy of a thick-the-box approach, particularly evident in university alliances. Each alliance draws back on specific values that are different from efficiency or budgetary constraints only, and they could cover topics such as sustainability, intercomprehension, arts, digitalization of learning, Europeanist sentiments, informality in learning, and many others. Consequently, papers on the operationalization of social impact assessment techniques in university alliances are still rare, as the process of operationalization requires intense work based on confrontation, discussion, and revision that puts together teaching, learning, and third mission activities with their expected long-term impacts. Therefore, this research fills a gap in the literature regarding the investigation of the positive effects of EUAs in the development of sustainable communities.

Previous studies are scarce not only because the phenomenon is relatively new, but also because the literature on social impact evaluation of research is still growing and expanding^16^. As such, our paper is contributing as herein a university alliance there are multi-levels of accountability that need to be explored^11^. A first level of accountability is established between the university partners of the alliance (as inner communities), as most of the impact is also generated through changes in their organizational and management practices, sometimes creating new divisions or new roles in existing directorates to manage the workload of the alliance. A second level of accountability is established between the university alliance towards the European Commission. This type of accountability, while crucial for making academic institutions responsible to the public government^59^, is less innovative in terms of the parties involved, as it is traditionally a responsibility towards the achievement of goals and performance included in the research project proposal. For instance, UNITA alliance has played a primary role in supporting the adoption by the European Commission of the need to introduce, for the first time, a European degree provided by the transnational alliances. This will have a tremendous impact on the academic careers of students, in the light of a more attractive and sustainable higher education sector. A third level of accountability is more inter-organizational as in this case, the university alliance has involved multiple-actors with multiple-values^60–62^, where societal impacts are co-created, like in the case of rural internships by involving rural communities, municipalities, business partners, students, professors, staff. This type of accountability is toward the external stakeholder of each university partnering with the alliance, as the success of the projects relies on the ability to engage communities and societies living in internal areas. In addition, we have noticed the existence of a further level of sub-politics of accountability. At first, between project coordinators and task leaders, towards all professors, researchers, and administrative staff members, who are part of the project in creating courses, developing rural mobilities, etc., without receiving direct financial compensation. Those taking part in the activities are working for a higher good that exceeds the traditional academic activities. Among these subjects, societal impact measurement acquired sense, especially in evaluating the contribution of the project in demonstrating its ability to promote sustainable communities, enhancing the academic profile, the knowledge or relationships co-developed, the self-esteem of project participants, and other intangible values, which would remain not visible if they were not measured^63^.

Regarding the *emic* side, it should be noted that this project has been developed to operationalize a process of social impact assessment to orient, monitor, and make tangible progress towards the specific social value creation objectives of the project itself, while complying with the principle of attribution. This must be done to avoid shifting “from attribution to contribution [..] as a way to reward the engagement of university research without having to attribute specific causal relationships to such complex challenges”^16^. During the operationalization phase, we noted a great and generalized interest among project participants in providing feedback and insights regarding the application of accountability logic and social impact assessment framework within UNITA. One of the reasons for such a collaborative environment is that within UNITO professors and researchers have completely different backgrounds (literature, ICT, media, agriculture, chemistry, physics, etc.), but without having specific skills in applying methods such as the Theory of Change or the Impact Pathway logic, and thus appreciated the external support. A second great motivator has been serving as the pioneers of the process of social impact assessment, aiming to generalize it and scale the method to the entire alliance. Specifically, the elaboration and testing of the KPIs for expressing outcomes and impacts has been intense, as it has let WP and task leaders become more aware of their responsibility to create transformational impacts on society and on the participants.

However, the process has not only resulted in positive things, as several barriers and resistance to change were found. At first, the idea of understanding the temporal and logical link between outputs-outcomes-impacts has been sometimes difficult to explain, as most research projects are usually focused on the monitoring of the results, without really asking “what happens next”. As such, the operationalization of the ToC has required to challenge the status quo of mainstream thinking, breaking geographical and temporal boundaries. On the geographical side, the university alliance should have an impact on all the territories directly involved, and the cooperation between partners is fundamental to map and track the impact generated. On the temporal side, the operationalization of the ToC has required those involved to think and envision “what should happen next”, having both a short-term timeframe (immediately after the project), as well as focusing on the long-lasting effect.

Previous research has predominantly concentrated on the contributions of individual universities towards the enhancement of sustainable development in their territories^64,65^. Studies have assessed the contribution of university courses in promoting local development^66^ fostering sustainable communities^67^, and nurturing future sustainability-conscious professionals by inspiring a deep curiosity in the new generation to further investigate these issues^2,68^. Beyond teaching, researchers have also explored how universities contribute to sustainable development in local communities through various activities such as capacity building^65^, civic engagement^17^, partnerships with local communities^69^, and collaboration with firms^70^. However, this body of research, while extensive, has overlooked the potential of institutionalized university collaboration networks, such as EUAs, in advancing sustainable communities^71^. Distinctively, our investigation pioneers in exploring the role that such alliances may play in promoting sustainable development within and beyond academic settings. This role encompasses various avenues, including the promotion and implementation of new sustainability educational programs, which studies suggest may support the responsible growth of local economies^72^. Additionally, it involves the potential contributions of EUAs in introducing new policies at the European level to foster sustainability and sustainable communities. Concurrently, the absence of a structured framework to evaluate these contributions represents a notable gap in the existing literature. Our study addresses this lacuna by proposing the first evaluative framework specifically designed to assess the effectiveness and impact of university alliances on sustainable community development. Thus, this research marks a significant advancement in the field of higher education sustainability, providing both a novel analytical perspective on the benefits of university alliances for the development of local sustainable communities and a methodological tool for their assessment.

As a case study adopting an interventionist approach contributing to the novel body of literature on university alliances, the limitations of this study can serve as a basis for future research. In particular, the contribution of this study is limited to the context studied^73^, thus the replication of this study on other alliances could unravel areas of improvement of our proposed impact assessment framework. Another limitation of this study is that two researchers were embedded in the organization with a participatory process. Although two outside academic advisors have been included to mitigate researchers’ bias, future research could be enriched with external interviews with the researchers to enrich data triangulation to inform the findings^74^. Additionally, this study does not interpret the results in light of established managerial and accounting theories, which can enrich the academic discourse on the social impacts and accountability issues involving EUAs. Future research could also investigate how social impact assessment practices affect public policies in higher education, particularly those related to forming new cross-border networks of academic and research institutions^6^ as catalysts for fostering sustainable communities. These contributions are poised to enrich academic discourse and offer practical insights for the strategic implementation of sustainability initiatives within the higher education sector.

### Supplementary Information

Supplementary Information.

## Data Availability

The dataset generated during and/or analyzed during the current study is available from the corresponding author upon reasonable request.
