# Supplementary material for: Developing a societal impact evaluation framework for sustainable European University Alliances
Source: Sci Rep. 2024 Jun 6;14:13052. doi: 10.1038/s41598-024-63933-9 (PMC11156667; doi:10.1038/s41598-024-63933-9)
Supplement: Supplementary file 1 — Supplementary Information. [file 41598_2024_63933_MOESM1_ESM.docx]

**UNITA’s mission, objectives and governance structure**

Originating from the European Universities Initiative, UNITA represents an ambitious endeavor aimed at fortifying collaborations among higher education establishments, giving rise to cohesive networks termed as “European Universities”. From east to west, the six universities that constitute the original line-up of the alliance are Universidade de Beira Interior (Portugal), Universidad de Zaragoza (Spain), Université de Pau et des Pays de l'Adour (France), Université Savoie Mont Blanc (France), University of Turin (Italy), and Universitatea de Vest din Timisoara (Romania). However, the alliance has been expanded in 2023, and it has welcomed six new partners: Instituto Politécnico da Guarda (Portugal), Universidad Pública de Navarra (Spain), Università degli Studi di Brescia (Italy), Universitatea Transilvania Brasov (Romania), Haute Ecole Spécialisée de Suisse Occidentale (Switzerland) and the Yuriy Fedkovych Chernivtsi National University (Ukraine). With the addition of the new partners, the students involved are 248,000 and the staff members are 20,000. The alliance also has 35 associated partners, including national and international organizations, local authorities, other universities, and representatives of the socio-economic sphere.

Committed to advancing pioneering pedagogical and research methodologies, UNITA members converge in their focus on Renewable Energy, Cultural Heritage, and Circular Economy domains. These thematic foci have been deliberately selected due to their profound influence on ecosystem resilience, rural advancement, and the enrichment of vocational prospects for both students and communities.

The mission of the alliance is expressed by 8 main objectives (UNITA website), around which its activities are structured:

1. Creating a participative, open, inclusive and effective European university.

2. Developing excellent research-based and student-centered education.

3. Promoting multilingualism and language diversity in Europe.

4. Reducing inequalities between central and non-core regions through the sustainable development of rural and mountainous areas.

5. Creating a stimulating learning environment.

6. Achieving mobility for all.

7. Contribute to strengthening European identity.

8. Ensuring the continuity and uptake of the UNITA approach.

UNITA’s governance structure strives to promote a democratic and participatory process, through a multi-level and multi-stakeholder model with strong interconnections among the decisional, consultative, operational, and administrative bodies of UNITA. The core decisional body, as well as the final decision maker, is the Governance Board, which is responsible for strategic decision-making, including the budget, and the overall direction of the project. The Governance Board is also supported by other three democratic advisory bodies: the Student’s Assembly, the Advisory Council and the Quality and Evaluation Board. The Governance Board is composed of a total of 15 members with voting rights among which there are the rectors of the member universities. During the first year of the project, each university has established a purposely created UNITA office for communication, administration and to manage citizen engagements and public participation in local events and activities. From an operational point of view, the activities of the alliance follow traditional project management techniques, with an architecture composed of 8 Work Packages (WP), each matching one of the alliance’s goals, led by WP task forces and coordinated by the UNITA Management Committee. The former oversees the implementation of activities, while the latter serves as the bridge between the decisional bodies and the WP activities.

**Data collection**

The hermeneutic unit of this study is summarized in Table 1, where both the three phases of the intervention and the types of interactions that occurred can be traced. Thus, it is emphasized that the type of information collected was diverse and complex, following a series of more or less structured moments, both in the degree of formality and in the type of participants, which are listed below in Supplementary Table 1, with a brief description.

Supplementary Table 1 - Data collection

| Date | Type of interaction | UNITA Participants | Duration |
| --- | --- | --- | --- |
| 1^st^ phase – Planning the intervention: development of the method | | | |
| 04/05/2022 | First interaction | UNITO rector | 30 min. |
| 18/05/2022 | Second interaction and confirmation of the project | 2 UNITA governance members | 30 min. |
| 06/07/2022 | Workshop | 4 Italian public university evaluation agency representatives, 3 University societal impact experts | 3 h. |
| 17/09/2022 | Informal intensive meeting | Student representative | 2 h. |
| 29/09/2022 | Workshop | 6 representatives of different European University alliances | 4 h. |
| 30/09/2022 | Focus group | 2 UNITA governance members | 1 h. 30 min. |
| 05/10/2022 | Focus meeting | UNITA executive coordinator | 1h. |
| 14/11/2022 | Informal intensive meeting (online) | UNITA WP leader | 30 min. |
| 2^nd^ phase – Implementation of the intervention: application and presentation of the method | | | |
| 25/11/2022 | Workshop | UNITO rector, 2 UNITA governance members, UNITA executive coordinator, 15 UNITA task leaders, 1 representative of UNITA student assembly | 2 h. |
| 5/12/2022 | Focus meeting (online) | External UNITA consultant, UNITA executive coordinator, 5 task leaders and 5 task vice-leaders | 1 h. |
| 19/12/2022 | Focus meeting (online) | UNITA executive coordinator, 5 task leaders and 5 task vice-leaders | 1 h. |
| 21/12/2022 | Focus meeting | 2 UNITA governance members, UNITA executive coordinator | 1 h. |
| 3^rd^ phase – Intervention evaluation: validation and feedback on the selected indicators | | | |
| 17/01/2023 | Workshop | 2 Italian public University evaluation agency representatives, 1 REF representative | 3 h. |
| 14/03/2023 | Informal intensive meeting | 1 UNITA administration staff representative | 30 min. |
| 14/03/2023 | Informal intensive meeting | 3 UNITA administrative staff representatives | 30 min. |
| 19/03/2023 | Impromptu interview | Student assembly representative | 1 h. |
| 20/03/2023 | Focus meeting | UNITA executive coordinator and UNITA Quality and Evaluation expert | 1 h. |
| 28/03/2023 | Field observation | UNITA governance member (UNITO vice-rector) | 30 min. |
| 26/04/2023 | Focus meeting | WP leader | 30 min. |
| 26/04/2023 | Focus meeting | WP leader | 30 min. |
| 08/05/2023 | Focus meeting | WP leader | 1 h. |
| 09/05/2023 | Focus meeting | WP leader | 1 h. 30 min. |
| 10/05/2023 | Focus meeting | WP leader | 30 min. |
